# Associations of Blood Lipid Traits with Cognitive-Related Outcomes and Their Sensitivity to APOE-Region Instrumental Variables: A Mendelian Randomization and Colocalization Study

**DOI:** 10.3390/genes17101218

**Published:** 2026-09-30

**Authors:** Xiaoya Tian, Xiaolin Xue

**Affiliations:** School of Traditional Chinese Medicine, Beijing University of Chinese Medicine, Beijing 102401, China; 20240931062@bucm.edu.cn

**Keywords:** Mendelian randomization, colocalization, blood lipids, brain-related outcomes

## Abstract

**Background:** Neurodegenerative, cognitive, and cerebrovascular outcomes are central to brain health. Previous studies have linked blood lipid traits to these outcomes, but associations differ across lipid components and outcome types. However, the contribution of APOE remains unclear. We compared these patterns, quantified their dependence on APOE, and identified shared signals outside the region. **Methods:** We used two-sample Mendelian randomization to examine associations between four blood lipid traits from the Global Lipids Genetics Consortium and seven cognitive-related outcomes from FinnGen R11, followed by regional colocalization analysis. We stratified instruments into APOE and non-APOE regions and compared region-specific estimates. Robust non-APOE signals supported by colocalization were further assessed using UK Biobank BIG40 imaging-derived phenotypes. **Results:** Genetically predicted high-density lipoprotein cholesterol (HDL-C) was inversely associated with the FinnGen composite endpoint of stroke excluding SAH and with cerebrovascular sequelae. Total cholesterol (TC) was positively associated with Alzheimer disease (AD) and dementia.Colocalization supported shared regional signals for TC-AD near TSBP1/TSBP1-AS1, LDL-C-AD within the extended HLA region, and HDL-C-stroke excluding SAH near ILRUN. Estimates for AD, dementia, and memory loss were sensitive to APOE-region exclusion, whereas the HDL-C-stroke estimate was materially unchanged. In separate exploratory analyses of 13 BIG40 imaging phenotypes, HDL-C colocalized with left lateral and right medial orbitofrontal gray–white matter intensity contrast. **Conclusions:** Lipid-related genetic effects differ across brain outcomes. AD and dementia estimates were sensitive to APOE-region instruments, whereas the HDL-C-associated signal for the composite stroke endpoint persisted after their exclusion. Exploratory imaging results nominate orbitofrontal intensity contrast as an HDL-C correlate.

## 1. Introduction

Blood lipid traits have been associated with stroke risk in epidemiological studies, including stroke outcomes that exclude subarachnoid hemorrhage [1,2]. The 2024 Lancet Commission on dementia prevention identified high low-density lipoprotein cholesterol (LDL-C) as a modifiable risk factor for dementia [3]. However, findings vary across lipid components and exposure periods. High total cholesterol (TC) in midlife has been associated with an increased risk of dementia later in life [4]. Similarly, higher LDL-C before the age of 65 has been linked to greater long-term dementia risk [5]. In contrast, higher high-density lipoprotein cholesterol (HDL-C) has been associated with a lower risk of late-onset Alzheimer disease (AD) in older populations [6]. A dose–response meta-analysis of prospective studies reported associations of higher TC and triglyceride (TG) levels with an increased risk of AD [7]. Collectively, these findings indicate that a single lipid measure or clinical outcome cannot adequately characterize the relationship between lipid traits and brain health. AD and dementia predominantly reflect neurodegenerative processes, whereas stroke and other cerebrovascular outcomes primarily represent vascular brain injury. Memory impairment reflects cognitive dysfunction at the phenotypic level. Treating these heterogeneous outcomes as manifestations of a single lipid–brain mechanism may obscure differences in their underlying genetic architecture and biological pathways.

Among the seven outcomes considered in this study, memory loss warrants separate consideration because it is the only symptom-defined outcome, whereas the others are disease diagnoses. The inclusion of memory loss as an outcome was based on three considerations. First, memory-related symptoms may be clinically relevant to early cognitive changes. A study found that older adults with subjective memory complaints experienced faster subsequent cognitive decline and had greater white matter hyperintensity volume [8]. Second, a memory-related outcome may complement dementia diagnoses by capturing cognitive changes before a clinical diagnosis is made. In a longitudinal study of individuals with preclinical AD, a decline in episodic memory test performance was detectable approximately four years before clinical diagnosis [9]. Analyzing memory loss separately from diagnosed dementia therefore allows us to examine whether associations with lipid traits are also observed for memory-related symptoms that may emerge earlier. Third, direct evidence links lipid levels to memory performance. In a longitudinal study of 13,258 US adults with repeated measurements over approximately 14 years, higher baseline high-density lipoprotein cholesterol (HDL-C) was associated with higher memory scores during follow-up [10]. Taken together, memory loss provides a symptom-level complement to the six disease-diagnosed outcomes, allowing comparison of lipid associations across memory-related symptoms and clinical diagnoses.

Complementing this symptom-defined outcome, the six disease-diagnosed outcomes encompass AD and dementia as well as vascular conditions relevant to cognitive impairment. Previous research has shown that both neurodegenerative pathology and cerebrovascular injury are associated with cognitive decline [11]. Progressive cognitive impairment is a central clinical feature of AD and dementia [11]. Vascular dementia (VaD) falls within the spectrum of vascular cognitive impairment, in which cognitive deficits are closely linked to cerebrovascular pathology [12,13]. Stroke and other cerebrovascular diseases may contribute to cognitive decline through acute brain injury, cerebral small-vessel disease, and chronic abnormalities in cerebral perfusion [14,15]. Sequelae of cerebrovascular disease capture longer-term effects of cerebrovascular events, among which persistent cognitive impairment is an important outcome [16,17]. Including these different types of outcomes also allows us to compare changes in lipid association estimates in sensitivity analyses of APOE-region genetic instruments. APOE ε4 is one of the strongest genetic risk factors for late-onset AD, making AD a key outcome for assessing the sensitivity of these associations to instruments in the APOE region [17]. Together, the selected outcomes provide a framework spanning AD-related dementia and vascular conditions associated with cognitive impairment. Memory loss and the six disease-diagnosed outcomes thus constitute seven outcomes relevant to cognitive impairment, covering both symptom-level and diagnostic outcomes, as well as neurodegenerative and cerebrovascular aspects of cognitive decline.

Evaluating lipid associations across these outcomes also requires attention to the genetic instruments used, particularly variants in the APOE region. Previous Mendelian randomization (MR) studies have reported inconsistent findings on associations between lipid traits and AD. One important source of heterogeneity is the treatment of the APOE region. Proitsi et al. excluded rs4420638 in the APOE region from the lipid genetic score. They found no association of genetically predicted LDL-C, HDL-C, TC, or TG with late-onset AD [18]. Østergaard et al. observed some evidence of lipid-related associations when the APOE-adjacent variant rs6857 was included [19]. However, these associations were no longer significant after variants strongly associated with AD were excluded. MR estimates of drug-target effects also vary according to the target gene and the variants used as instruments [20,21]. Studies of HDL subfractions address a different biological question from analyses of total HDL-C, and some excluded variants in the APOE region a priori [22]. These analytical choices are particularly important because APOE participates in both peripheral and central nervous system lipid transport. It is also the strongest genetic risk locus for late-onset AD and is linked to amyloid deposition, tau-related neurodegeneration, and cerebrovascular function [23,24]. Estimates that include the APOE region may therefore capture both lipid-related genetic effects and direct AD-related effects arising from this locus, complicating the interpretation of lipid–AD associations. Comparing estimates with and without these variants can help assess the sensitivity of observed associations to the APOE region and clarify differences across studies. Three interrelated gaps remain in the current evidence. First, most studies focus on AD or dementia as primary outcomes. Few have examined neurodegenerative, cognitive, and cerebrovascular outcomes within the same analytical framework, limiting direct comparisons across outcome categories. Second, previous studies have mainly reported overall MR estimates. Few have directly compared estimates obtained with and without the APOE region under the same analytical criteria. Consequently, the extent to which these associations depend on the APOE region has not been systematically quantified. Third, disease-related genetic signals are rarely integrated with brain structural phenotypes. Cerebral small-vessel disease involves endothelial dysfunction, blood–brain barrier disruption, ischemic injury, inflammation, and myelin damage [25]. White matter hyperintensities (WMHs) are associated with an increased risk of stroke, dementia, and cognitive decline [26]. Late-life cognitive impairment may also reflect the combined effects of AD pathology and cerebral microvascular injury [27]. Large-scale imaging-genetic resources, such as Oxford BIG40, provide an opportunity to connect disease-related genetic signals with quantifiable features of brain structure [28]. Research should therefore extend beyond estimating overall associations between lipid traits and AD to comparing APOE dependence across brain outcomes. Accordingly, analyses across multiple brain outcomes, together with comparisons that include and exclude APOE-region variants, may clarify which observed lipid associations are sensitive to the APOE region. Examining shared genetic signals outside this region may further identify loci relevant to lipid traits, cerebrovascular outcomes, and brain structural phenotypes.

This study integrated lipid GWAS summary statistics from the Global Lipids Genetics Consortium (GLGC), clinical outcome data from FinnGen, brain imaging GWAS data from Oxford BIG40, and trait annotations from the GWAS Catalog. We first used two-sample Mendelian randomization and regional colocalization to examine the associations of four lipid traits (HDL-C, LDL-C, TC, and TG) with seven cognitive and cerebrovascular disease or symptom outcomes (Alzheimer disease, dementia, vascular dementia, stroke excluding subarachnoid hemorrhage, other cerebrovascular diseases, sequelae of cerebrovascular disease, and memory loss). We then separated genetic instruments by their location within or outside a prespecified APOE region and compared the resulting estimates to assess their sensitivity to this region. Finally, we evaluated supported signals outside the APOE region against brain imaging phenotypes using regional colocalization analysis.

## 2. Materials and Methods

### 2.1. Study Design and Data Sources

**Figure 1:**
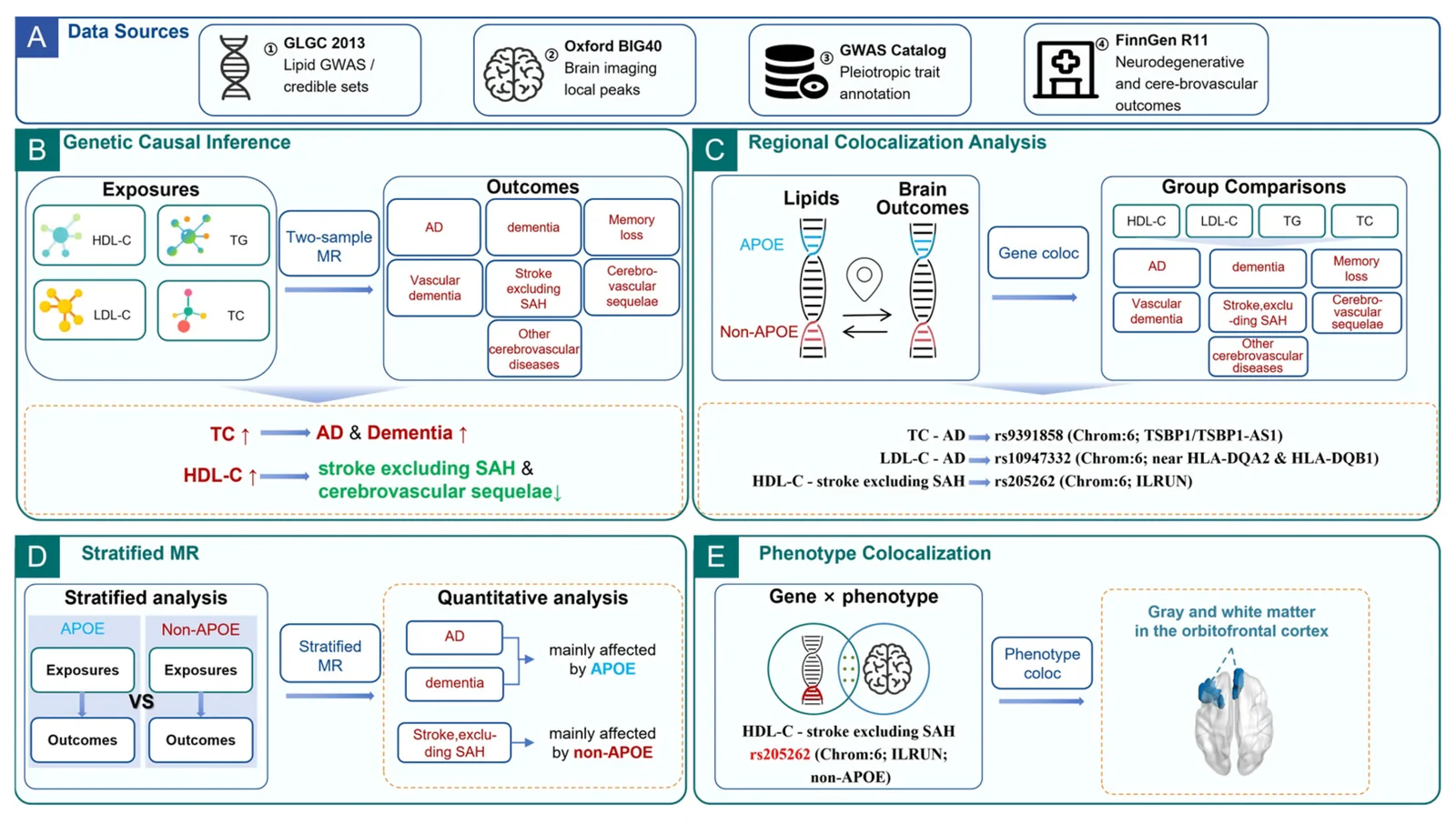
Study framework.

We used a multistage genetic analysis framework. First, we conducted two-sample MR and regional colocalization analyses across brain-related outcomes using HDL-C, LDL-C, TC, and TG as exposures. Second, APOE-stratified MR was performed to assess the extent to which the observed associations depended on the APOE region. Finally, we conducted colocalization analyses with brain imaging phenotypes for lipid–cerebrovascular signals that remained supported outside the APOE region. The overall study workflow is illustrated in Figure 1.

All data were obtained from publicly available GWAS summary statistics and imaging-genetic resources; no new participants were recruited. GLGC GWAS summary statistics were used to construct genetic instruments for lipid traits [29]. Oxford BIG40 [28] provided genetic association data for brain imaging-derived phenotypes (IDPs) for subsequent imaging-genetic analyses. The GWAS Catalog was used to annotate genetic loci and their associated traits [30]. Clinical outcomes were defined using GWAS summary statistics from FinnGen Release 11 (R11) [31].

All four lipid exposures were obtained exclusively from the GLGC 2013 reported by Willer et al. The meta-analysis included up to 188,577 participants of European ancestry. Because the sample size varied across markers, Table 1 reports the largest marker-specific analytical N for each trait.

Within contributing cohorts, lipid measurements were adjusted for age, age squared, and sex. The residuals were quantile-normalized to a standardized inverse-normal scale. Published β coefficients represent the change in this transformed phenotype per effect allele. Thus, each MR estimate corresponds to a 1-SD increase in the transformed exposure, not a concentration change in mg/dL or mmol/L. We used the published summary statistics without further phenotype transformation or individual-level covariate adjustment.

We selected seven FinnGen R11 endpoints from 2440 phenotypes to compare lipid associations across dementia-related diagnoses, vascular brain disease and its consequences, and a registry-recorded memory symptom. The endpoints were G6_ALZHEIMER, F5_DEMENTIA, F5_VASCDEM, I9_STR, I9_CEREBDOTH, I9_SEQULAE, and MEMLOSS.

Among them, G6_ALZHEIMER used ICD-10 code G30 and ICD-9 code 3310. F5_DEMENTIA used ICD-10 codes F0[0–3], ICD-9 codes 290, 3310, or 4378A, and ICD-8 code 290; it also incorporated KELA reimbursement code 307 and anti-dementia medication purchases (ATC N06D), included F5_ALZHDEMENT, F5_VASCDEM, F5_DEMINOTH, and F5_DEMNAS, and required at least three events. F5_VASCDEM used ICD-10 code F01 or ICD-9 code 4378. I9_STR used ICD-10 codes I61, I63 (excluding I63.6), or I64; ICD-9 codes 431, 4330A, 4331A, 4339A, 4340A, 4341A, 4349A, or 436; and ICD-8 codes 431, 433, 434, or 436, excluding 43101 and 43191. This composite endpoint includes intracerebral hemorrhage, cerebral infarction, and stroke not specified as hemorrhage or infarction; it is therefore not specific to ischemic stroke. I9_CEREBDOTH used ICD-10 codes I67.8, I67.9, or I68.8; ICD-9 codes 436, 4371, or 4378X; and ICD-8 code 438. I9_SEQULAE used ICD-10 code I69 or ICD-9 code 438. MEMLOSS used ICD-10 codes R41.1-R41.3 and represents a registry-recorded memory symptom, not an objective cognitive test. FinnGens endpoint-specific control exclusions were applied: F5_DEMENTIA excluded F5_ORGANIC; F5_VASCDEM excluded F5_DEMENTIA; I9_STR excluded I9_STR_EXH and I9_TIA; I9_CEREBDOTH and I9_SEQULAE excluded I9_CEREBVASC; and MEMLOSS excluded F5_DEMENTIA, G6_ALZHEIMER, and G6_AD_WIDE.

They comprised neurodegenerative outcomes (AD and dementia), cerebrovascular outcomes (vascular dementia, stroke excluding subarachnoid hemorrhage [SAH], other cerebrovascular diseases, and sequelae of cerebrovascular disease), and a registry-recorded memory symptom (memory loss). The FinnGen endpoint codes and case and control numbers are presented in Table 2.

### 2.2. Genetic Instrument Selection and Two-Sample MR

We used two-sample Mendelian randomization (MR) to evaluate associations between genetically predicted lipid traits and brain-related outcomes. The analysis was conducted in accordance with the STROBE-MR guidelines. For details, please refer to the Supplementary File S6. The GLGC 2013 and FinnGen R11 GWAS may partially overlap through Finnish legacy cohorts, including FINRISK, Finnish twin cohorts, and NFBC1986. Sample sizes, ancestry, phenotype definitions, and data sources for all GWAS datasets are summarized in Supplementary File S1.

GWAS summary statistics for HDL-C, LDL-C, TC, and TG from the GLGC lipid study were used as exposure data. Genetic instruments were initially selected at the genome-wide significance threshold of $p<5\times 10^{-8}$ and an instrument-strength criterion of $F=(\beta_{exposure}/SE_{exposure}{)}^{2}>10$. Before LD clumping, variants had to have valid GRCh37 coordinates and be present in the European ancestry subset (*n* = 503) of the 1000 Genomes Project Phase 3 reference panel. Exposure alleles were matched directly or by strand complement. Variants with <95% nonmissing genotypes were excluded, and remaining missing genotypes were mean-imputed.

For each lipid trait, variants were ranked by *p* value and greedily clumped within a 10 Mb window, retaining a variant only if its r^2^ with every more significant retained variant was <0.001. Residual same-chromosome pairs with r^2^ ≥ 0.001 were recorded in the supplementary LD tables. Clumping yielded 303 trait-specific SNP–exposure records (87 HDL-C, 76 LDL-C, 86 TC, and 54 TG), representing 251 unique rsIDs. Detailed information on the 303 LD-clumped instruments is provided in the Supplementary File S2.

For each exposure–outcome pair, records were merged by rsID and harmonized through allele matching, strand correction, and alignment of effect allele directions. Palindromic SNPs with missing exposure allele frequencies or frequencies within 0.42–0.58 were excluded to avoid bias arising from ambiguous strand orientation. The harmonized instruments were used to calculate the Wald ratio and its standard error for each SNP. Each outcome analysis retained 292 harmonized SNP–exposure records (82 HDL-C, 75 LDL-C, 82 TC, and 53 TG).

The primary analysis used multiplicative random-effects inverse-variance-weighted (IVW) MR. The IVW point estimate was calculated using inverse-variance weights. When Cochran’s Q exceeded its degrees of freedom (the number of instruments minus one), we increased the standard error to account for excess heterogeneity; otherwise, the standard error was left unchanged. This adjustment does not change the point estimate but can widen its confidence interval and increase its *p* value. Fixed-effect IVW estimates are reported as Supplementary results. To account for multiple testing, the Benjamini–Hochberg procedure was applied across all 28 primary MR tests, corresponding to four lipid traits and seven prespecified brain-related outcomes. A false discovery rate (FDR)-adjusted *p* value < 0.05 was considered statistically significant.

### 2.3. Regional Colocalization Analysis

Regional colocalization analysis was performed using GLGC lipid GWAS and FinnGen brain-related outcome GWAS data to assess whether paired traits shared genetic signals within specific genomic regions. Across the four lipid traits and seven FinnGen outcomes, we evaluated 95 exposure–outcome-specific candidate region windows. For each of the 28 exposure–outcome pairs, up to three candidate windows were selected around instrument SNPs ranked by outcome-association *p* value, with exposure-association *p* value used as a secondary ranking criterion. An additional window overlapping the predefined APOE interval was included when an eligible instrument was available. Each window extended 500 kb upstream and downstream of its lead SNP. Identical windows within the same exposure–outcome pair were deduplicated; partially overlapping but nonidentical windows were retained and analyzed separately. SNPs falling within overlapping windows were included in each corresponding analysis. All 95 windows contained at least 25 SNPs with summary statistics available for both traits.

Colocalization posterior probabilities were calculated from regional SNP-level marginal summary statistics using an approximate Bayes factor (ABF) framework. The per-SNP prior probabilities were set to $p_{1}=1\times 10^{-4},\;p_{2}=1\times 10^{-4},\;p_{12}=1\times 10^{-5}$, corresponding to associations with the exposure, the outcome, and both traits, respectively. The prior variances for exposure and outcome effect sizes were set to 0.15^2^ and 0.20^2^, respectively. Accordingly, posterior probabilities for these regions are conditional on the single-causal-variant-per-trait model. Colocalization was evaluated using five mutually exclusive hypotheses. PP.H3 denotes the posterior probability that two distinct causal variants drive the two traits within the same region. PP.H4 denotes the posterior probability that both traits share a causal variant. A high PP.H3 suggests independent genetic association signals within the region, whereas a high PP.H4 supports a shared genetic signal. We prespecified PP.H4 ≥ 0.80 as the threshold for evidence of a shared regional genetic signal.

### 2.4. APOE-Region-Stratified MR

To assess whether lipid–brain outcome associations depended on signals within the APOE region, we stratified the genetic instruments using a predefined genomic interval. Instruments were classified as APOE-region or non-APOE instruments according to the genomic position of the exposure-associated variant.

The APOE region was predefined as chromosome 19:44.0–46.5 Mb on the GRCh37 reference genome, encompassing APOE and its surrounding extended linkage disequilibrium (LD) block. The boundaries were based on APOE-region definitions used in previous large-scale AD GWASs [32].

LD clumping retained 10 regional instruments: HDL-C: rs2075650 and rs2288912; LDL-C: rs445925, rs157580, and rs17800760; TC: rs7412, rs75687619, and rs8103315; and TG: rs439401 and rs3760627. For HDL-C, rs2288912 was excluded because it is a C/G palindromic SNP with an exposure allele frequency of 0.4987, leaving rs2075650 as the sole eligible APOE-interval instrument. Among the 292 harmonized SNP–exposure records per FinnGen R11 outcome, 283 were outside and 9 were within the predefined APOE interval. The other retained APOE-interval counts were three for LDL-C, three for TC, and two for TG.

MR estimates were recalculated for each instrument group and prespecified outcome. The multiplicative random-effects IVW method was used when a subgroup contained two or more instruments; the Wald ratio was used when only one instrument was available. Estimates based on fewer than five instruments were interpreted as having limited robustness.

Differences between APOE-region and non-APOE effect estimates were assessed using Cochran’s Q statistic with 1 degree of freedom. We also calculated the proportion of the total inverse-variance weight contributed by APOE-region instruments. This was defined as the sum of inverse-variance weights for APOE-region instruments divided by the sum of weights for all instruments. The metric quantified the relative contribution of the APOE region to the overall IVW estimate.

### 2.5. Sensitivity Analyses Methods

Sensitivity analyses were conducted for all 28 exposure–outcome combinations comprising four lipid traits and seven neurological outcomes. Analyses used both the full instrument set and a restricted set excluding variants within the APOE region (non-APOE instruments). We assessed heterogeneity, directional horizontal pleiotropy, and the influence of individual instruments, and compared estimates across complementary MR methods.

Cochran’s Q test was used to assess heterogeneity among variant-specific effect estimates. Directional horizontal pleiotropy was assessed using the MR-Egger intercept test. The weighted median method was used as a complementary estimator. This method provides consistent estimates when at least 50% of the total analytical weight is contributed by valid instruments. Leave-one-out analyses sequentially excluded each instrument and recalculated the IVW estimate to assess the influence of individual variants. Effect estimates are reported as odds ratios (ORs) with 95% confidence intervals (CIs).

### 2.6. Colocalization Methods for Brain Imaging Phenotype 

To examine potential brain-imaging correlates of lipid–cerebrovascular associations, we performed colocalization analyses with brain imaging phenotypes for exposure–outcome pairs meeting four criteria. These were (1) statistical significance in MR after correction for multiple testing; (2) stable effect direction and magnitude after excluding APOE-region instruments; (3) regional colocalization evidence for a shared signal (PP.H4 ≥ 0.80); and (4) a shared locus outside the APOE region. Based on these prespecified criteria, the association between HDL-C and stroke excluding SAH was selected for brain imaging-genetic analysis, with rs205262 as the lead variant.

To explore potential brain imaging pathways linking blood lipids and cerebrovascular outcomes, we used IDP GWAS summary statistics from the UK Biobank BIG40 brain imaging genetics study. A region extending 500 kb upstream and downstream of rs205262 was defined (chr6:34,063,164–35,063,164, hg19), and genetic association data for IDPs within this interval were extracted. We evaluated regional colocalization of BIG40 IDPs with HDL-C and stroke excluding SAH separately.

We used the 13 BIG40 IDPs as a focused, exploratory panel to examine candidate structural correlates of the rs205262 HDL-C-stroke locus. They comprised orbitofrontal gray–white matter intensity contrast, caudate and pallidum volumes, FA/MD measures in the genu of the corpus callosum and right posterior limb of the internal capsule, and white-matter hyperintensity (WMH) volume.

We included WMH burden and tract-specific diffusion measures as candidate cerebrovascular phenotypes: prior work has reported associations between tract-specific white-matter IDPs and stroke [33], while lacunar-stroke studies have examined infarcts in the posterior limb of the internal capsule and WMH as small-vessel-disease features [34]. The orbitofrontal and caudate/pallidum measures were complementary exploratory phenotypes; we did not assume that each was an established stroke marker. This panel was not a comprehensive screen of all 3935 BIG40 IDPs.

For the separate rs205262–IDP association screen, *p* values were Bonferroni-adjusted across the 13 IDPs (adjusted *p* = min[13 × P, 1]). For regional colocalization, each IDP was assessed against both HDL-C and stroke, yielding 26 pairwise comparisons. Because colocalization produces posterior probabilities rather than frequentist *p* values, no formal FDR correction was applied.

For each IDP–reference trait pair, summary statistics were merged by rsID, and effect directions were aligned by matching effect alleles. Palindromic SNPs and variants with incompatible alleles were excluded. Colocalization analysis was performed only when at least 50 shared SNPs were available in both GWAS datasets. An ABF framework was used to calculate posterior probabilities for the five colocalization hypotheses. The prior effect-size variance was set to 0.15^2^ for HDL-C and quantitative IDPs and to 0.20^2^ for the binary stroke outcome. The per-SNP prior probabilities were set to $p_{1}=1\times 10^{-4},\;p_{2}=1\times 10^{-4},\;p_{12}=1\times 10^{-5}$. We prespecified PP.H4 ≥ 0.80 as the threshold for evidence of a shared regional genetic signal between an IDP and a reference trait.

### 2.7. Software and Reproducibility

Primary summary-statistics processing, allele harmonization, Mendelian randomization, multiple-testing correction, APOE-region stratification, LD assessment, and visualization were performed with Python 3.12.4 using NumPy 1.26.4, pandas 2.2.2, SciPy 1.13.1, statsmodels 0.14.2, Matplotlib 3.8.4, seaborn 0.13.2, requests 2.32.2, openpyxl 3.1.2, and Patsy 0.5.6. The complete analysis scripts are provided with the reproducibility materials. All analyses used the parameter values and random seeds recorded in the corresponding run metadata.

## 3. Results

### 3.1. Two-Sample MR

**Figure 2:**
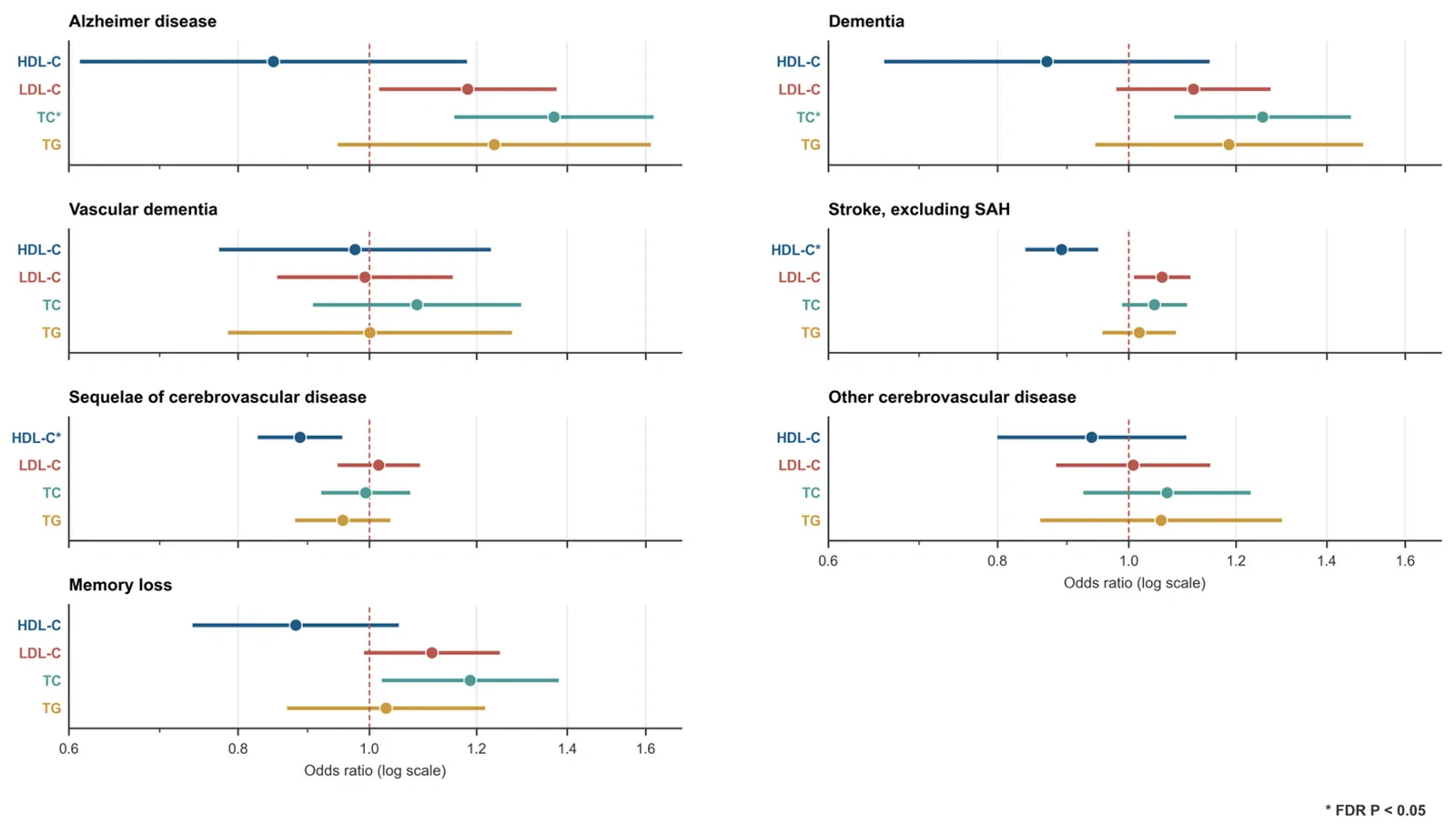
MR effect estimation of lipid traits and brain outcomes.

Two-sample MR was first used to evaluate directional associations between lipid traits and brain outcomes. The results are presented in Figure 2.

Using multiplicative random-effects IVW, HDL-C-associated genetic variation was inversely associated with stroke excluding SAH (OR = 0.89, *p* < 0.05; FDR *p* < 0.05) and sequelae of cerebrovascular disease (OR = 0.89, *p* < 0.05; FDR *p* < 0.05). TC-associated genetic variation was positively associated with AD (OR = 1.37, *p* < 0.05; FDR *p* < 0.05) and dementia (OR = 1.26, *p* < 0.05; FDR *p* < 0.05). Complete MR results for all lipid–brain outcome pairs are presented in Table A1.

### 3.2. Colocalization Analysis

**Figure 3:**
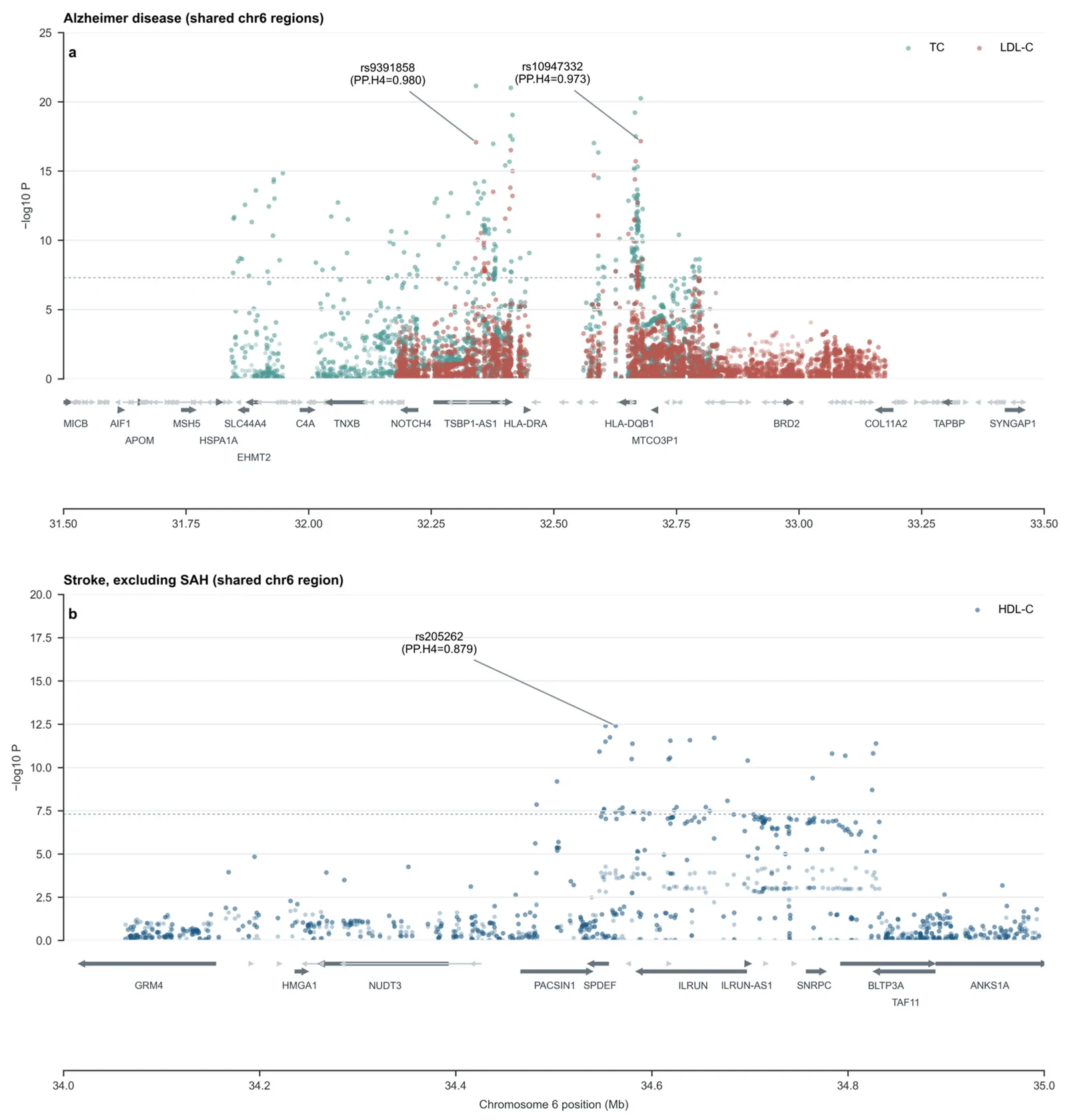
Results of regional colocalization of lipid traits and brain outcomes. (**a**) TC and LDL-C association signals with AD. (**b**) HDL-C association signals with stroke excluding SAH. Arrows indicate the labeled lead variants.

Regional colocalization analysis assessed whether lipid traits and brain-related outcomes shared genetic signals within specific genomic regions. The results are presented in Figure 3 and Table 3.

TC and AD showed a strong shared genetic signal near rs9391858 on chromosome 6 in the TSBP1/TSBP1-AS1 region (PP.H4 = 0.980). LDL-C and AD shared a signal near rs10947332 on chromosome 6 in the HLA region, adjacent to HLA-DQA2 and HLA-DQB1 (PP.H4 = 0.973). HDL-C and stroke excluding SAH shared a regional genetic signal near rs205262 on chromosome 6, an intronic variant in ILRUN (PP.H4 = 0.879). No other evaluated region reached PP.H4 ≥ 0.80. Complete results are presented in the Supplementary File S5 and Table A2.

### 3.3. APOE-Region vs. Non-APOE-Region MR Estimates

**Figure 4:**
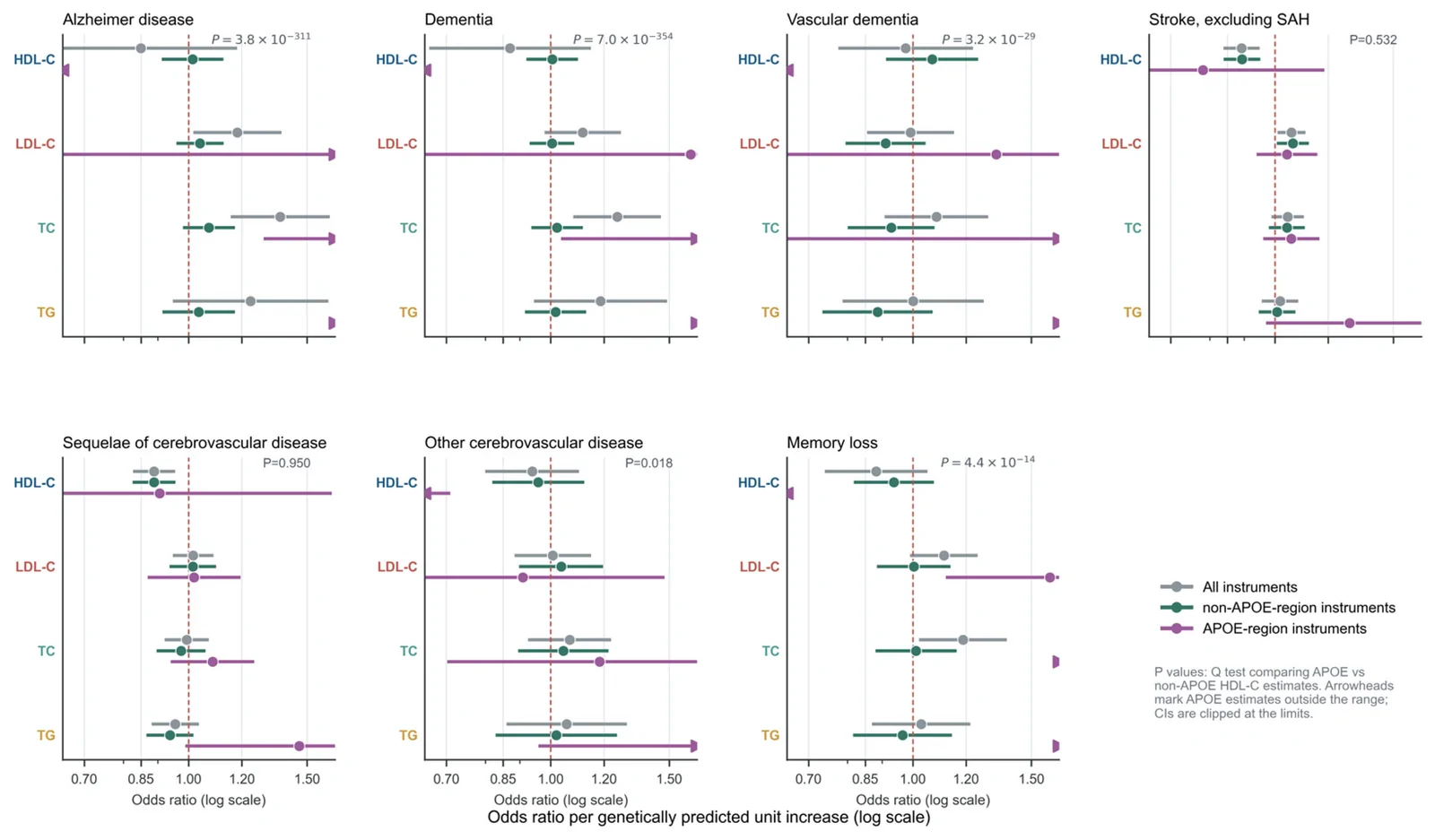
APOE and non-APOE-stratified MR estimates.

We further conducted an APOE-region-stratified sensitivity analysis, partitioning genetic instruments into APOE-region and non-APOE groups. The results are presented in Figure 4.

For AD and dementia, all-instrument estimates generally attenuated towards the null after APOE-region instruments were excluded. For HDL-C, the all-instrument ORs were 0.849 for AD and 0.870 for dementia, whereas the corresponding non-APOE ORs were 1.014 and 1.005. The differences between regional estimates were statistically significant for both outcomes (*p* < 0.001). TC showed a similar pattern, with all-instrument ORs of 1.369 for AD and 1.255 for dementia, compared with 1.072 and 1.022 after APOE-region exclusion. The corresponding between-region *p* values were 0.026 and 0.048. TG estimates also differed significantly between regions for both outcomes (*p* < 0.001), whereas the LDL-C differences were not statistically significant (*p* = 0.470 for AD and *p* = 0.489 for dementia).

For vascular dementia, strong regional heterogeneity was observed for HDL-C and TG (*p* < 0.001 for both). Some APOE-region estimates were extreme, whereas the corresponding non-APOE estimates were generally close to the null. Regional differences were not statistically significant for LDL-C (*p* = 0.493) or TC (*p* = 0.223).

For memory loss, the all-instrument estimates for LDL-C and TC moved towards the null after APOE-region exclusion. The LDL-C estimate changed from an OR of 1.112 to 1.003, and the TC estimate changed from 1.187 to 1.011. Between-region differences were observed for HDL-C (*p* < 0.001), LDL-C (*p* = 0.016), TC (*p* < 0.001), and TG (*p* < 0.001).

For sequelae of cerebrovascular disease, regional estimates were similar for HDL-C, LDL-C, and TC. The corresponding between-region *p* values were 0.950, 0.965, and 0.203, respectively. The non-APOE HDL-C association remained significant (OR = 0.889, *p* = 0.00150, FDR *p* = 0.0210), whereas the non-APOE confidence intervals for LDL-C and TC included the null. TG showed nominal evidence of a regional difference (*p* = 0.030), but the non-APOE confidence interval also included the null.

For stroke excluding SAH, the HDL-C estimate remained stable after APOE-region exclusion. The all-instrument estimate was OR = 0.892 (*p* = 3.03 × 10^−4^, FDR *p* = 0.00424), compared with OR = 0.893 (*p* < 0.001, FDR *p* < 0.05) using non-APOE instruments. The difference between regional estimates was not statistically significant (*p* = 0.532). The corresponding regional comparisons for LDL-C, TC, and TG were also not statistically significant (*p* = 0.736, 0.795, and 0.096, respectively). These findings indicate that the HDL-C-associated signal for stroke excluding SAH was not materially dependent on the predefined APOE-region instruments. Complete stratified results are presented in Table A3.

### 3.4. Sensitivity Analyses

**Figure 5:**
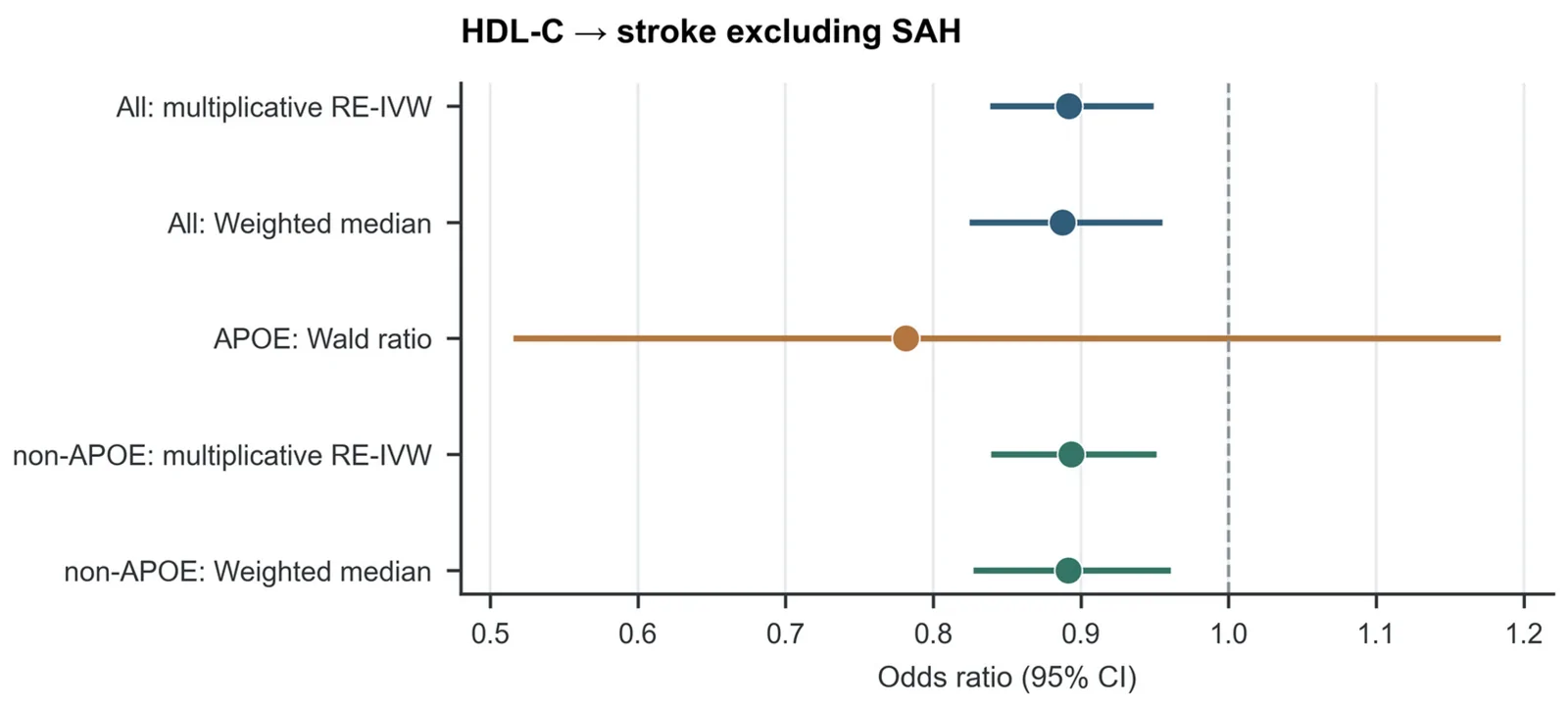
Sensitivity estimates for HDL-C → stroke excluding SAH.

For HDL-C and stroke excluding SAH, the multiplicative random-effects IVW estimate remained similar after APOE-region exclusion (all instruments: OR = 0.892, 95% CI 0.838–0.949, FDR *p* = 0.00424; non-APOE instruments: OR = 0.893, 95% CI 0.839–0.951, FDR *p* = 0.0119). The corresponding weighted-median estimates also indicated inverse associations (OR = 0.887 and 0.891). MR-Egger intercept tests did not indicate directional pleiotropy (*p* = 0.186 and 0.191), although Cochran’s Q indicated heterogeneity in both analyses (*p* < 0.001). Leave-one-out estimates remained inverse after each SNP was removed (OR ranges: 0.886–0.901 and 0.887–0.903, respectively; Figure 5). Complete sensitivity and numerical leave-one-out results for all exposure–outcome combinations are provided in Supplementary Files S3 and S4, respectively.

### 3.5. Brain Imaging Phenotype Colocalization

Two-sample MR, regional colocalization, and APOE-region-stratified analyses showed that genetically predicted higher HDL-C was associated with a reduced risk of stroke excluding SAH (OR = 0.89). Regional colocalization identified a shared signal for HDL-C and stroke excluding SAH near rs205262 on chromosome 6 (PP.H4 = 0.879). rs205262 is an intronic variant in ILRUN.

The same lead variant, rs205262, was observed for HDL-C. Among the 13 targeted imaging-derived phenotypes, HDL-C showed evidence of colocalization with MRI-derived gray–white matter intensity contrast in the left lateral orbitofrontal region (PP.H4 = 0.903) and the right medial orbitofrontal region (PP.H4 = 0.803). Complete colocalization results for BIG40 IDPs are presented in Table A4.

## 4. Discussion

This study compared the associations of four lipid traits with seven cognitive and cerebrovascular outcomes using MR, regional colocalization, and exploratory imaging-genetic analyses. The results differed by lipid trait and outcome: TC showed the clearest primary associations with AD and dementia, whereas the inverse association of HDL-C with stroke excluding SAH persisted after removal of APOE-region instruments. These patterns warrant separate consideration of outcome heterogeneity, sensitivity to the APOE region, and evidence from non-APOE loci.

### 4.1. Associations of Lipid Traits with Different Cognitive-Related Outcomes

For neurodegenerative outcomes, higher genetically predicted TC was associated with AD and dementia in the primary multiplicative random-effects IVW analysis. The LDL-C estimate was positive for AD but did not survive FDR correction, and its association with dementia was not statistically significant. Associations of HDL-C and TG with these outcomes should likewise be interpreted in light of the multiple-testing results and the regional sensitivity analyses below. Although AD and dementia share cognitive impairment as a defining feature, the broader dementia category includes heterogeneous causes. Registry-recorded memory loss is a symptom-defined outcome rather than a diagnosis of AD or dementia; concordance of the regional patterns across these outcomes does not establish a shared disease mechanism. Previous studies have reported variable associations between lipid traits and AD or dementia [35,36,37].

For cerebrovascular outcomes, genetically predicted higher HDL-C was associated with lower risks of stroke excluding SAH and sequelae of cerebrovascular disease. Higher LDL-C showed a nominal positive association with stroke excluding SAH that did not survive FDR correction. These outcome-specific differences suggest that the lipid association observed for the broad stroke endpoint should not be assumed to apply equally to AD, dementia, or memory loss. Prior genetic evidence concerning HDL-C and atherosclerotic cardiovascular disease offers context [38], although stroke excluding SAH encompasses more than ischemic stroke and is not a direct replication of that phenotype.

### 4.2. Sensitivity of Lipid–Outcome Associations to APOE-Region Instruments

Excluding APOE-region instruments substantially attenuated several MR estimates for AD and dementia. For TC, the resulting ORs were 1.072 for AD and 1.022 for dementia. The LDL-C ORs decreased from 1.182 to 1.040 for AD and from 1.116 to 1.004 for dementia; the non-APOE estimates remained slightly above one, but their confidence intervals included the null. The HDL-C point estimate for AD shifted from an OR of 0.849 to 1.014, whereas positive estimates for TC and TG were also attenuated. Together with the observed patterns for registry-recorded memory loss, these changes indicate sensitivity of the corresponding lipid–outcome associations to inclusion of APOE-region instruments. They do not quantify a biological contribution of APOE or establish mediation by lipid levels.

The APOE region contained one HDL-C instrument, three LDL-C instruments, three TC instruments, and two TG instruments. APOE-region instruments accounted for 22.0% and 22.2% of the inverse-variance weight in the LDL-C analyses of AD and dementia, respectively. The single HDL-C instrument in this region accounted for approximately 1.5% of the weight in the AD analysis, yet its exclusion changed the pooled OR from 0.849 to 1.014. Thus, the change in an estimate after regional exclusion cannot be inferred from the regional weight alone. Weighted-median estimates for several AD and dementia comparisons were directionally concordant but weaker than IVW estimates, further tempering causal interpretation [39,40].

By comparison, the inverse association between HDL-C and stroke excluding SAH was stable when APOE-region instruments were removed. The weighted-median ORs were 0.887 (95% CI 0.824–0.955) using all instruments and 0.891 (95% CI 0.827–0.961) after regional exclusion. The MR-Egger intercept test did not provide evidence of directional horizontal pleiotropy, and leave-one-out estimates remained within a narrow range. Cochran’s Q nevertheless indicated heterogeneity, so agreement among these analyses supports robustness to the specified exclusions without eliminating other sources of bias. No statistically significant difference between APOE and non-APOE estimates was found for the reported associations of HDL-C with stroke excluding SAH and its sequelae, or of LDL-C with stroke excluding SAH.

Regional colocalization adds a distinct perspective. The APOE locus spans a gene-dense region including BCAM, NECTIN2, TOMM40, APOE, and APOC1, with complex linkage disequilibrium and haplotype structure [41,42]. For TC and AD, APOE-region colocalization favored distinct association signals (PP.H3 > 0.99) over a shared signal (PP.H4 < 0.001); the same H3-dominant pattern appeared for lipid trait pairs involving AD, dementia, and registry-recorded memory loss. This is compatible with substantial sensitivity of MR estimates to regional instruments: MR and colocalization address different questions, and an estimate that changes after excluding a region need not imply a shared causal variant there [43].

### 4.3. Imaging Phenotypes and APOE-Region Sensitivity

The imaging analysis helps interpret signals outside the APOE region, where the HDL-C association with stroke excluding SAH remained stable. Non-APOE colocalization supported shared regional signals for TC and AD near rs9391858 (PP.H4 = 0.980), LDL-C and AD near rs10947332 (PP.H4 = 0.973), and HDL-C and stroke excluding SAH near rs205262 at the ILRUN locus (PP.H4 = 0.879). Previous work has implicated ILRUN in lipoprotein production, plasma lipid regulation, and atherosclerosis [44]. These results show that attenuation of pooled AD estimates after APOE-region exclusion does not rule out individual non-APOE regions with colocalization support.

At the rs205262 region, HDL-C also colocalized with left lateral and right medial orbitofrontal gray–white matter intensity contrast (PP.H4 = 0.903 and 0.803, respectively). This exploratory imaging result identifies a regional genetic overlap involving HDL-C and brain imaging phenotypes. It does not establish that the imaging traits mediate the HDL-C-stroke association, nor does it identify a causal variant or effector gene. Because imaging follow-up here focused on a selected non-APOE signal, these findings do not provide a direct comparison of APOE-region and non-APOE-region effects on brain structure.

### 4.4. Limitations and Future Directions

Several limitations affect interpretation. First, MR requires instruments that are relevant to the exposure, independent of confounders, and free from pathways to the outcome outside the exposure. Horizontal pleiotropy and genetic overlap among lipid traits may bias univariable estimates; the absence of an MR-Egger intercept signal does not prove that all instruments satisfy these assumptions. Second, MR reflects lifelong genetically proxied differences in lipid traits, whose estimates should not be equated with the effects of treatment initiated at a particular age [45,46]. The broad registry definitions of dementia, stroke excluding SAH, and memory loss also limit disease-specific interpretation.

Third, the predefined APOE interval includes multiple genes and potentially distinct association signals. Its trait-specific regional analyses contained only one to three instruments after harmonization. Regional estimates can therefore be unstable, and changes after exclusion should be interpreted as sensitivity to the selected instrument set rather than evidence for a discrete APOE-mediated effect. Fourth, the single-signal colocalization model may be restrictive in complex regions such as APOE, the extended MHC, and ILRUN. Replication in independent cohorts with more precise outcome definitions, conditional and fine-mapping analyses, and multiple-signal colocalization could help resolve these loci. Multivariable MR could examine the extent to which the cerebrovascular association assigned to HDL-C is shared with other lipid traits; imaging and functional studies would then be needed to investigate the biological pathways suggested by the genetic results [47].

## 5. Conclusions

We used two-sample MR to compare four lipid traits across seven outcomes and found that genetically predicted higher HDL-C was associated with lower risks of stroke excluding SAH and cerebrovascular sequelae, whereas higher TC was associated with greater risks of AD and dementia. We then excluded APOE-region instruments in sensitivity analyses and found that the AD and dementia estimates attenuated, while the HDL-C-stroke excluding SAH estimate remained similar.

We used regional colocalization to identify a shared signal for HDL-C and stroke excluding SAH near rs205262, outside the APOE region. We further analyzed Oxford BIG40 imaging data and found colocalization between HDL-C and orbitofrontal gray–white matter intensity contrast at this locus, but no corresponding colocalization between stroke and the imaging phenotype.

We conclude that blood lipid associations differ across outcomes and in their sensitivity to APOE-region instruments. We identified rs205262 as a candidate locus for further study of lipid-related cerebrovascular risk, and the imaging finding remains exploratory.

## Figures and Tables

**Table 1:** Characteristics of the GLGC lipid exposure GWASs.

Exposure	GLGC Release	Ancestry	Largest Marker-Specific Analytical N	Genome Build
HDL-C	2013	European	187,167	GRCh37/hg19
LDL-C	2013	European	173,082	GRCh37/hg19
TC	2013	European	187,365	GRCh37/hg19
TG	2013	European	177,861	GRCh37/hg19

**Table 2:** FinnGen R11 brain-related outcomes and sample sizes.

Outcome	FinnGen Endpoint	Cases	Controls
Alzheimer disease	G6_ALZHEIMER	11,755	441,978
Dementia	F5_DEMENTIA	20,667	428,053
Vascular dementia	F5_VASCDEM	3116	433,066
Stroke, excluding SAH	I9_STR	30,521	408,788
Sequelae of cerebrovascular disease	I9_SEQULAE	14,063	412,105
Other specified cerebrovascular diseases/ cerebrovascular disorders in diseases classified elsewhere	I9_CEREBDOTH	2331	412,105
Memory loss	MEMLOSS	3013	430,206

**Table 3:** Results of regional colocalization of lipid traits and brain outcomes.

Exposure	Outcome	Lead SNP	Region/Gene	PP.H4
TC	AD	rs9391858	TSBP1-AS1	0.980273042
LDL-C	AD	rs10947332	MTCO3P1	0.973076451
HDL-C	Stroke, excluding SAH	rs205262	ILRUN	0.879200256

**Figure A1:**
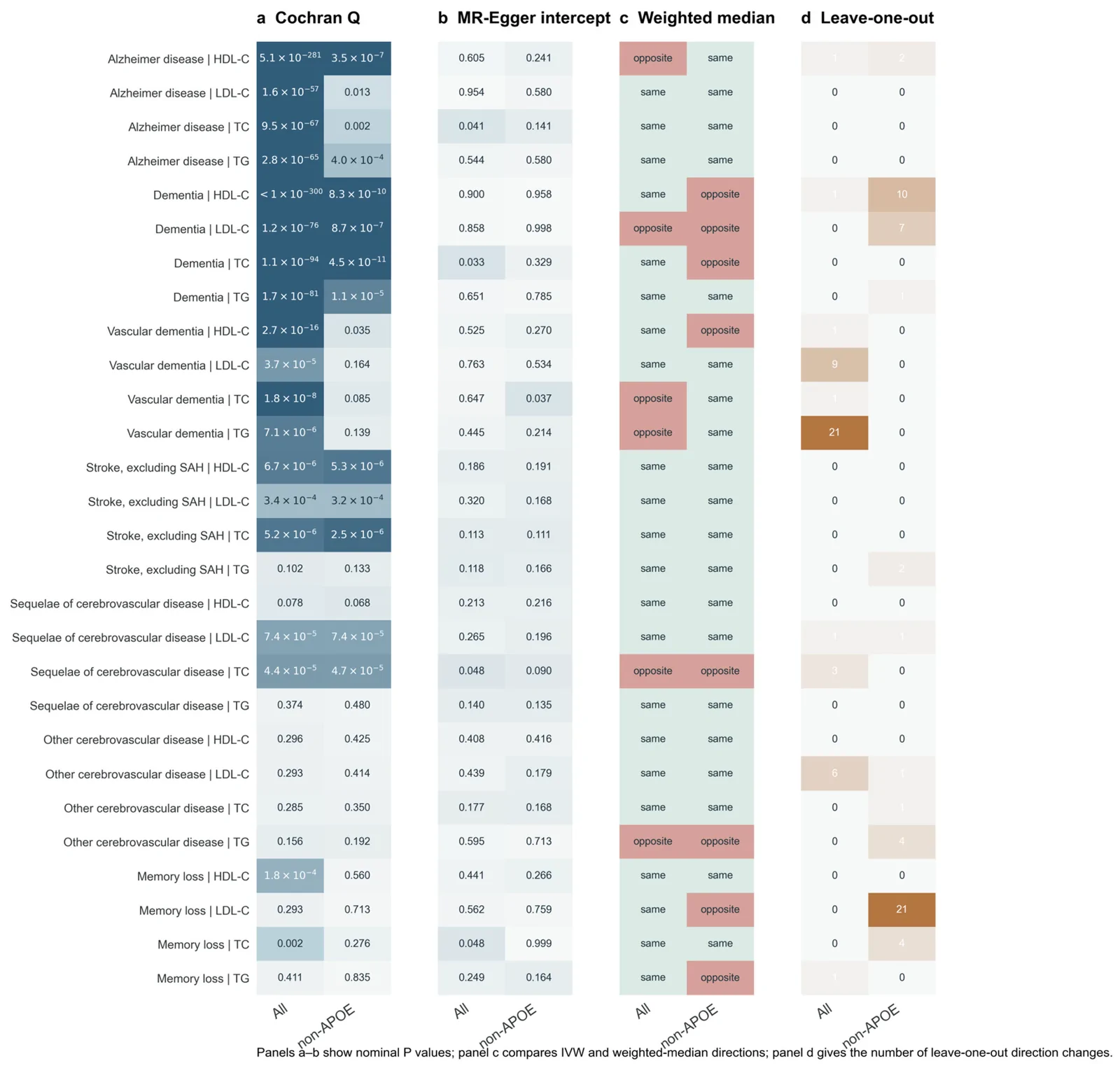
Overview of sensitivity analyses across all 28 exposure–outcome combinations.

**Figure A2:**
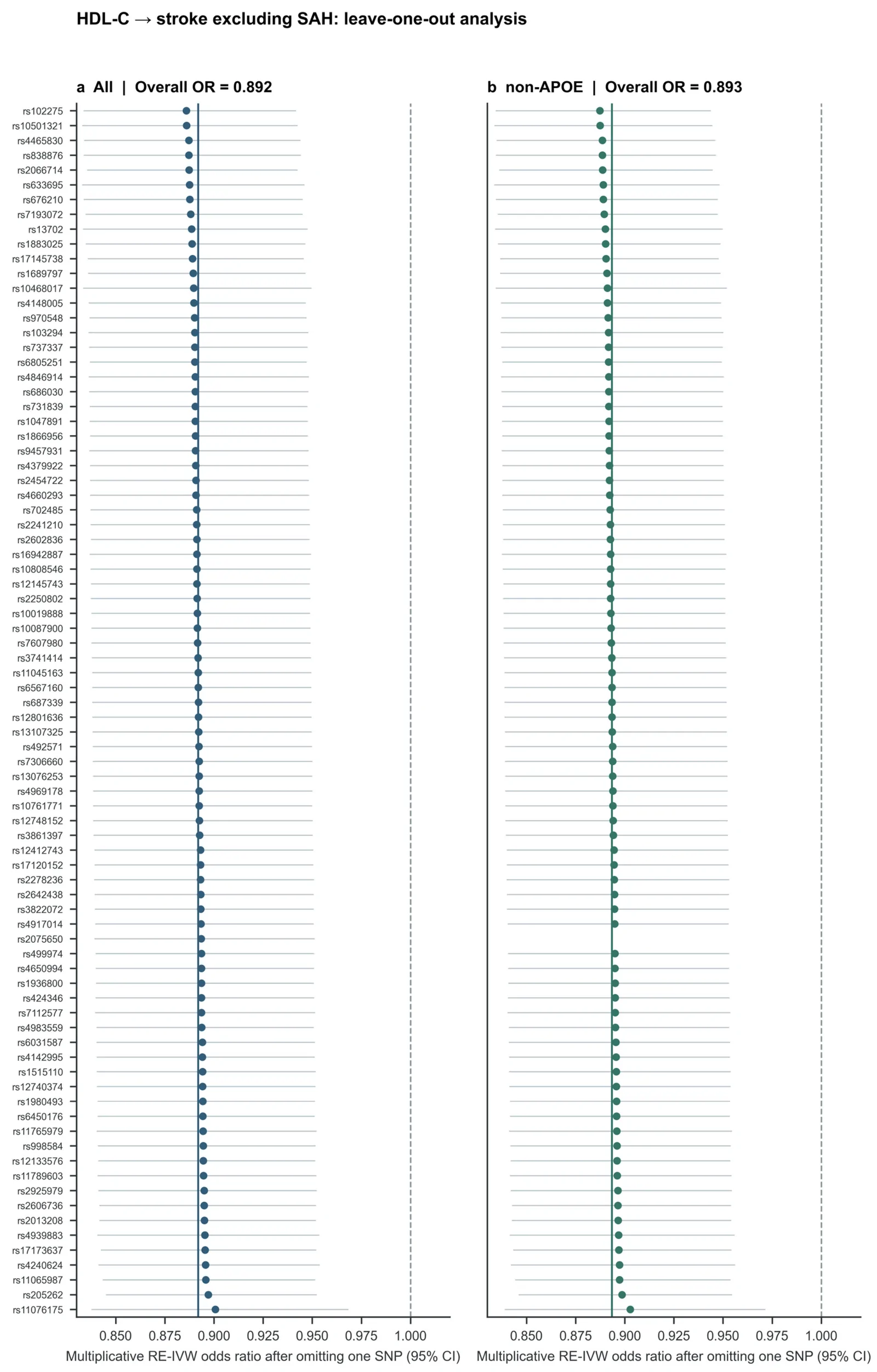
Leave-one-out analysis of the association between HDL-C and stroke excluding subarachnoid hemorrhage (SAH), based on all instruments and non-APOE-region instruments.

## Data Availability

All datasets used in this study are publicly available from GLGC 2013: https://csg.sph.umich.edu/abecasis/public/lipids2013 (accessed on 5 August 2026), FinnGen R11 https://finngen.gitbook.io/documentation/r11/data-download (accessed on 5 August 2026), Oxford BIG40 https://open.oxcin.ox.ac.uk/ukbiobank/big40/ (accessed on 5 August 2026), and the GWAS Catalog: https://www.ebi.ac.uk/gwas (accessed on 5 August 2026). Analysis code and report-related materials are available at https://github.com/XeniaTian/dcp_obstudy (accessed on 1 September 2026).

## Supplementary Materials

The following supporting information can be downloaded at: https://www.mdpi.com/article/10.3390/genes17101218/s1, Supplementary File S1: GWAS dataset inventory, pairwise sample-overlap assessments, overlap summary, and references; Supplementary File S2: Characteristics of the 303 LD-clumped instruments, including APOE-region SNPs; Supplementary File S3: Full MR results for the 28 lipid–outcome combinations in the all-instrument and non-APOE analyses; Supplementary File S4: Complete leave-one-out results for the prespecified lipid–outcome analyses; Supplementary File S5: Full regional colocalization results, SNP-level posterior probabilities, and gene annotations; Supplementary File S6: Completed STROBE-MR reporting checklist.

## Abbreviations

The following abbreviations are used in this manuscript:

ABF	Approximate Bayes factor
AD	Alzheimer disease
APOE	Apolipoprotein E
BIG40	Oxford Brain Imaging Genetics 40k-subject release
CI	Confidence interval
FA	Fractional anisotropy
FDR	False discovery rate
FIRST	FMRIB’s Integrated Registration and Segmentation Tool
FUT2	Fucosyltransferase 2
GLGC	Global Lipids Genetics Consortium
GWAS	Genome-wide association study
HDL-C	High-density lipoprotein cholesterol
HLA	Human leukocyte antigen
IDP/IDPs	Imaging-derived phenotype/imaging-derived phenotypes
ILRUN	Inflammation and lipid regulator with UBA-like and NBR1-like domains
IV	Instrumental variable
IVW	Inverse-variance-weighted
LD	Linkage disequilibrium
LDL-C	Low-density lipoprotein cholesterol
MR	Mendelian randomization
OR	Odds ratio
PP.H3	Posterior probability that the two traits are associated with distinct causal variants
PP.H4	Posterior probability that the two traits share a causal variant
R11	FinnGen Release 11
SAH	Subarachnoid hemorrhage
SNP/SNPs	Single-nucleotide polymorphism/single-nucleotide polymorphisms
STROBE-MR	Strengthening the Reporting of Observational Studies in Epidemiology using Mendelian Randomization
TBSS	Tract-based spatial statistics
TC	Total cholesterol
TG	Triglycerides

## Appendix A

This appendix provides the complete MR and colocalization results supporting the analyses presented in the main text.

Table A1 presents the full MR estimates, Table A2 summarizes the full regional colocalization results, Table A3 presents the APOE- and non-APOE-region MR estimates, and Table A4 reports the colocalization results for the BIG40 imaging-derived phenotypes.

Figure A1 and Figure A2 summarize the sensitivity analyses across all exposure–outcome combinations and the leave-one-out analysis for the HDL-C-stroke excluding SAH association using all instruments and non-APOE-region instruments.

**Table A1:** Full MR results for all lipid traits and brain outcomes pairs. Bold values indicate statistically significant results after FDR correction (adjusted *p* < 0.05).

Outcome	Exposure	OR (95% CI)	*p*	FDR *p*
AD	HDL-C	0.85(0.61–1.18)	0.331	0.579
	LDL-C	1.18(1.02–1.37)	**0.03**	0.118
	TC	1.37(1.16–1.62)	**<0.001**	**0.004**
	TG	1.24(0.95–1.61)	0.118	0.313
Dementia	HDL-C	0.87(0.66–1.15)	0.324	0.579
	LDL-C	1.12(0.98–1.27)	0.101	0.313
	TC	1.26(1.08–1.46)	**0.003**	**0.021**
	TG	1.19(0.94–1.49)	0.143	0.333
Vascular dementia	HDL-C	0.98(0.77–1.23)	0.836	0.955
	LDL-C	0.99(0.85–1.15)	0.92	0.955
	TC	1.08(0.91–1.29)	0.37	0.579
	TG	1.00(0.79–1.27)	0.994	0.994
Stroke excluding SAH	HDL-C	0.89(0.84–0.95)	**<0.001**	**0.004**
	LDL-C	1.06(1.01–1.11)	**0.021**	0.117
	TC	1.04(0.99–1.10)	0.123	0.313
	TG	1.02(0.96–1.08)	0.586	0.802
Sequelae of cerebrovascular disease	HDL-C	0.89(0.83–0.96)	**0.001**	**0.012**
	LDL-C	1.02(0.95–1.09)	0.656	0.835
	TC	0.99(0.92–1.07)	0.869	0.955
	TG	0.96(0.88–1.04)	0.273	0.546
Other cerebrovascular disease	HDL-C	0.94(0.80–1.10)	0.442	0.651
	LDL-C	1.01(0.88–1.15)	0.914	0.955
	TC	1.07(0.93–1.23)	0.372	0.579
	TG	1.06(0.86–1.30)	0.601	0.802
Memory loss	HDL-C	0.88(0.74–1.05)	0.161	0.347
	LDL-C	1.11(0.99–1.25)	0.071	0.248
	TC	1.19(1.02–1.38)	**0.025**	0.118
	TG	1.03(0.87–1.22)	0.74	0.901

**Table A2:** Full regional colocalization results. Bold values indicate evidence of colocalization (PP.H4 ≥ 0.80).

Region	Outcome	Exposure	Lead SNP	PP.H4	PP.H3	Shared SNPs
APOE	AD	HDL-C	rs2075650	<0.001	1	627
		HDL-C	rs2288912	<0.001	1	584
		LDL-C	rs157580	<0.001	1	620
		LDL-C	rs17800760	<0.001	1	707
		TC	rs7412	<0.001	1	611
		TC	rs75687619	<0.001	1	623
		TG	rs3760627	<0.001	1	565
		TG	rs439401	<0.001	1	607
	Dementia	HDL-C	rs2075650	<0.001	1	627
		HDL-C	rs2288912	<0.001	1	584
		LDL-C	rs157580	<0.001	1	620
		TC	rs7412	<0.001	1	611
		TC	rs75687619	<0.001	1	623
		TG	rs3760627	<0.001	1	565
		TG	rs439401	<0.001	1	607
	Memory loss	HDL-C	rs2075650	<0.001	1	627
		LDL-C	rs157580	<0.001	1	620
		LDL-C	rs445925	<0.001	1	604
		TC	rs7412	<0.001	1	611
		TC	rs75687619	<0.001	1	623
		TG	rs439401	<0.001	1	607
	Other cerebrovascular diseases	HDL-C	rs2075650	0.249	0.019	627
		LDL-C	rs157580	0.031	0.024	620
		TC	rs75687619	0.031	0.024	623
		TG	rs439401	0.048	0.023	607
	Sequelae of cerebrovascular disease	HDL-C	rs2288912	0.009	0.017	584
		LDL-C	rs157580	0.018	0.017	620
		TC	rs75687619	0.018	0.017	623
		TG	rs439401	0.039	0.017	607
	Stroke, excluding SAH	HDL-C	rs2288912	0.011	0.028	584
		LDL-C	rs157580	0.016	0.026	620
		TC	rs7412	0.016	0.027	611
		TG	rs3760627	0.013	0.026	565
	Vascular dementia	HDL-C	rs2075650	<0.001	1	627
		LDL-C	rs157580	<0.001	1	620
		TC	rs7412	<0.001	1	611
		TC	rs75687619	<0.001	1	623
		TC	rs8103315	<0.001	1	658
		TG	rs439401	<0.001	1	607
non-APOE	AD	HDL-C	rs2241210	0.509	0.037	1065
		LDL-C	rs10947332	**0.973**	0.025	2416
		TC	rs9391858	**0.98**	0.018	1806
		TG	rs2043085	0.001	0.88	1216
	Dementia	HDL-C	rs1980493	0.421	0.554	1847
		LDL-C	rs7832643	0.617	0.036	286
		LDL-C	rs6882076	0.543	0.422	1010
		TC	rs6882076	0.542	0.422	1013
		TG	rs6882076	0.483	0.477	1011
	Memory loss	HDL-C	rs4240624	0.253	0.118	1611
		HDL-C	rs2241210	0.097	0.041	1065
		LDL-C	rs9987289	0.252	0.118	1597
		TC	rs9987289	0.247	0.119	1606
		TG	rs2247056	0.156	0.145	2168
		TG	rs10501321	0.101	0.087	924
	Other cerebrovascular diseases	HDL-C	rs1515110	0.259	0.065	999
		HDL-C	rs17120152	0.032	0.069	1134
		LDL-C	rs11563251	0.439	0.084	1484
		LDL-C	rs6511720	0.284	0.036	461
		LDL-C	rs3798220	0.019	0.091	1336
		TC	rs11563251	0.401	0.094	1486
		TC	rs2287623	0.268	0.03	999
		TC	rs3798220	0.018	0.091	1340
		TG	rs2972146	0.369	0.055	990
		TG	rs3198697	0.194	0.005	124
		TG	rs10501321	0.158	0.145	924
	Sequelae of cerebrovascular disease	HDL-C	rs11065987	0.752	0.023	671
		HDL-C	rs17173637	0.156	0.239	893
		HDL-C	rs2066714	0.01	0.06	1349
		LDL-C	rs3780181	0.608	0.015	1587
		LDL-C	rs3184504	0.584	0.038	632
		LDL-C	rs267733	0.516	0.091	362
		TC	rs3184504	0.614	0.035	631
		TC	rs3780181	0.608	0.015	1593
		TC	rs2066714	0.009	0.06	1349
		TG	rs2247056	0.228	0.098	2168
		TG	rs287621	0.2	0.017	798
		TG	rs4587594	0.047	0.044	1171
	Stroke, excluding SAH	HDL-C	rs205262	**0.879**	0.042	597
		HDL-C	rs11065987	0.646	0.01	671
		HDL-C	rs11076175	0.019	0.042	850
		LDL-C	rs3184504	0.575	0.012	632
		LDL-C	rs6511720	0.501	0.022	461
		LDL-C	rs2315065	0.297	0.062	1272
		TC	rs3184504	0.569	0.012	631
		TC	rs2315065	0.568	0.038	1273
		TC	rs2814982	0.406	0.206	598
		TG	rs174535	0.074	0.02	765
		TG	rs998584	0.055	0.091	585
		TG	rs2247056	0.038	0.044	2168
	Vascular dementia	HDL-C	rs10501321	0.384	0.151	928
		HDL-C	rs4650994	0.229	0.022	570
		LDL-C	rs2710642	0.211	0.03	610
		LDL-C	rs6504872	0.063	0.073	573
		TG	rs10501321	0.34	0.162	924
		TG	rs731839	0.187	0.047	810

**Table A3:** Full APOE and non-APOE-stratified MR estimates. Bold values indicate statistically significant results (*p* < 0.05).

Outcome	Exposure	All-IV OR	APOE-Region OR	APOE	Non-APOE OR	Non-APOE	APOE-Region	Cochran’s Q
		(95% CI)	(95% CI)	SNPs	(95% CI)	SNPs	Weight (%)	*p*
AD	HDL-C	0.849	7.77 × 10^−6^	1	1.014	81	1.50%	**1421.81**
		(0.611–1.180)	(4.25 × 10^−6^–1.42 × 10^−5^)		(0.913–1.126)			**<0.001**
	LDL-C	1.182	1.86	3	1.04	72	22.04%	0.521
		(1.017–1.374)	(0.385–8.994)		(0.959–1.128)			0.47
	TC	1.369	5.021	3	1.072	79	15.81%	**4.95**
		(1.155–1.621)	(1.293–19.495)		(0.981–1.173)			**0.026**
	TG	1.237	87.919	2	1.035	51	4.00%	**69.3**
		(0.947–1.614)	(31.124–248.354)		(0.915–1.172)			**<0.001**
Dementia	HDL-C	0.87	3.78 × 10^−5^	1	1.005	81	1.42%	**1618.51**
		(0.660–1.147)	(2.32 × 10^−5^–6.17 × 10^−5^)		(0.921–1.098)			**<0.001**
	LDL-C	1.116	1.614	3	1.004	72	22.22%	0.48
		(0.979–1.272)	(0.422–6.170)		(0.931–1.084)			0.489
	TC	1.255	3.542	3	1.022	79	16.54%	**3.91**
		(1.080–1.459)	(1.036–12.111)		(0.936–1.117)			**0.048**
	TG	1.186	45.849	2	1.017	51	4.03%	**107.1**
		(0.944–1.489)	(22.460–93.595)		(0.916–1.129)			**<0.001**
Vascular dementia	HDL-C	0.976	1.09 × 10^−3^	1	1.068	81	1.31%	**125.91**
		(0.775–1.229)	(3.29 × 10^−4^–3.58 × 10^−3^)		(0.913–1.250)			**<0.001**
	LDL-C	0.992	1.331	3	0.911	72	22.63%	0.47
		(0.855–1.152)	(0.454–3.898)		(0.794–1.044)			0.493
	TC	1.084	2.23	3	0.929	79	17.69%	1.49
		(0.908–1.294)	(0.550–9.048)		(0.801–1.077)			0.223
	TG	1.001	17.435	2	0.887	51	4.07%	**47.3**
		(0.786–1.274)	(7.619–39.894)		(0.734–1.070)			**<0.001**
Stroke excluding SAH	HDL-C	0.892	0.781	1	0.893	81	1.21%	0.39
		(0.838–0.949)	(0.516–1.184)		(0.839–0.951)			0.532
	LDL-C	1.058	1.042	3	1.063	72	22.24%	0.114
		(1.009–1.111)	(0.939–1.156)		(1.006–1.123)			0.736
	TC	1.044	1.058	3	1.042	79	17.84%	0.068
		(0.988–1.104)	(0.961–1.164)		(0.979–1.108)			0.795
	TG	1.018	1.291	2	1.007	51	4.12%	2.76
		(0.956–1.083)	(0.970–1.718)		(0.946–1.073)			0.096
Cerebrovascular sequelae	HDL-C	0.889	0.906	1	0.889	81	1.21%	0.004
		(0.827–0.955)	(0.502–1.633)		(0.826–0.956)			0.95
	LDL-C	1.016	1.019	3	1.015	72	22.09%	0.002
		(0.947–1.090)	(0.869–1.195)		(0.937–1.099)			0.965
	TC	0.994	1.086	3	0.975	79	17.76%	1.62
		(0.921–1.072)	(0.941–1.252)		(0.896–1.060)			0.203
	TG	0.956	1.46	2	0.938	51	4.11%	**4.74**
		(0.881–1.036)	(0.989–2.156)		(0.866–1.017)			**0.03**
Other cerebrovascular disease	HDL-C	0.939	0.173	1	0.958	81	1.20%	**5.59**
		(0.800–1.102)	(0.042–0.710)		(0.819–1.122)			**0.018**
	LDL-C	1.007	0.909	3	1.036	72	21.76%	0.258
		(0.884–1.148)	(0.560–1.477)		(0.897–1.197)			0.611
	TC	1.067	1.182	3	1.044	79	17.50%	0.2
		(0.925–1.230)	(0.702–1.989)		(0.895–1.219)			0.655
	TG	1.056	2.422	2	1.019	51	4.11%	3.19
		(0.860–1.298)	(0.959–6.120)		(0.828–1.255)			0.074
Memory loss	HDL-C	0.882	9.21 × 10^−3^	1	0.937	81	1.30%	**57**
		(0.740–1.051)	(2.80 × 10^−3^–0.030)		(0.817–1.075)			**<0.001**
	LDL-C	1.112	1.598	3	1.003	72	22.18%	**5.84**
		(0.991–1.249)	(1.119–2.282)		(0.885–1.138)			**0.016**
	TC	1.187	2.746	3	1.011	79	16.06%	**20.64**
		(1.021–1.380)	(1.826–4.129)		(0.880–1.162)			**<0.001**
	TG	1.029	4.517	2	0.966	51	4.11%	**13.11**
		(0.869–1.218)	(1.994–10.232)		(0.815–1.144)			**<0.001**

**Table A4:** Full colocalization results of BIG40 IDP.

Code	idp	Reference_ Trait	n_shared	PP.H3	PP.H4	top_shared_rsid
1378	wg_lh_intensity-contrast_lateralorbitofrontal	HDL_C	519	0.0386	0.9027	rs205262
1604	IDP_dMRI_TBSS_MD_Genu_of_corpus_callosum		519	0.1773	0.8093	rs2814944
1415	wg_rh_intensity-contrast_medialorbitofrontal		519	0.0408	0.8029	rs205262
1413	wg_rh_intensity-contrast_lateralorbitofrontal		519	0.0624	0.687	rs205262
1380	wg_lh_intensity-contrast_medialorbitofrontal		519	0.0326	0.4489	rs205262
1620	IDP_dMRI_TBSS_MD_Posterior_limb_of_internal_capsule_R		519	0.0135	0.1088	rs2814944
0018	IDP_T1_FIRST_right_pallidum_volume		519	0.0275	0.0932	rs205262
1454	IDP_dMRI_TBSS_FA_Genu_of_corpus_callosum		519	0.0561	0.0932	rs2814944
0017	IDP_T1_FIRST_left_pallidum_volume		519	0.018	0.0639	rs205262
1471	IDP_dMRI_TBSS_FA_Posterior_limb_of_internal_capsule_R		519	0.0859	0.0184	rs205262
1437	IDP_T2_FLAIR_BIANCA_WMH_volume		519	0.0147	0.0125	rs2814944
0014	IDP_T1_FIRST_right_caudate_volume		519	0.0099	0.0115	rs2814944
0013	IDP_T1_FIRST_left_caudate_volume		519	0.0088	0.0076	rs2814944
1604	IDP_dMRI_TBSS_MD_Genu_of_corpus_callosum	Stroke, excluding SAH	515	0.0906	0.6705	rs2814985
1378	wg_lh_intensity-contrast_lateralorbitofrontal		515	0.0463	0.6084	rs2814985
1415	wg_rh_intensity-contrast_medialorbitofrontal		515	0.0453	0.2677	rs205262
1413	wg_rh_intensity-contrast_lateralorbitofrontal		515	0.0436	0.262	rs2814944
1380	wg_lh_intensity-contrast_medialorbitofrontal		515	0.0152	0.1353	rs2814985
1454	IDP_dMRI_TBSS_FA_Genu_of_corpus_callosum		515	0.0176	0.0424	rs2814985
1620	IDP_dMRI_TBSS_MD_Posterior_limb_of_internal_capsule_R		515	0.0043	0.0387	rs2814944
0018	IDP_T1_FIRST_right_pallidum_volume		515	0.0087	0.0244	rs205262
0017	IDP_T1_FIRST_left_pallidum_volume		515	0.0056	0.0164	rs205262
1437	IDP_T2_FLAIR_BIANCA_WMH_volume		515	0.0043	0.0065	rs2744967
1471	IDP_dMRI_TBSS_FA_Posterior_limb_of_internal_capsule_R		515	0.0247	0.0061	rs12206298
0014	IDP_T1_FIRST_right_caudate_volume		515	0.0029	0.004	rs2814944
0013	IDP_T1_FIRST_left_caudate_volume		515	0.0026	0.0025	rs9469864
