# Supplementary material for: Associations of Blood Lipid Traits with Cognitive-Related Outcomes and Their Sensitivity to APOE-Region Instrumental Variables: A Mendelian Randomization and Colocalization Study
Source: Genes (Basel). 2026 Sep 30;17(10):1218. doi: 10.3390/genes17101218 (PMC13704162; doi:10.3390/genes17101218)
Supplement: Supplementary file 1 [file genes-17-01218-s001.zip › Supplementary File S6.pdf]

STROBE-MR checklist of recommended items to address in reports of Mendelian randomization studies[1 2]

Item No.	Section		Checklist item	Page No.	Relevant text from manuscript
1	**TITLE and ABSTRACT**		Indicate Mendelian randomization (MR) as the study's design in the title and/or the abstract if that is a main purpose of the study	1	The title identifies the design as "A Mendelian Randomization and Colocalization Study"; the abstract describes two-sample MR.
	**INTRODUCTION**				
2	**Background**		Explain the scientific background and rationale for the reported study. What is the exposure? Is a potential causal relationship between exposure and outcome plausible? Justify why MR is a helpful method to address the study question	2–4	The Introduction describes four lipid traits, seven outcomes, the rationale for APOE-region sensitivity analyses, and the exploratory imaging analysis (§1).
3	**Objectives**		State specific objectives clearly, including pre-specified causal hypotheses (if any). State that MR is a method that, under specific assumptions, intends to estimate causal effects	4	The final Introduction paragraph states the objectives: two-sample MR and regional colocalization, APOE-region sensitivity, and imaging follow-up.
	**METHODS**				
4	**Study design and data sources**		Present key elements of the study design early in the article. Consider including a table listing sources of data for all phases of the study. For each data source contributing to the analysis, describe the following:	4–9; Tables 1–2	§2.1 outlines the workflow, and Figure 1 summarizes it. GLGC 2013, FinnGen R11, Oxford BIG40, and GWAS Catalog are identified. Tables 1–2 and the accompanying GWAS inventory workbook summarize sample sizes, ancestry, phenotype definitions, and data sources. §2.2 reports potential cohort-level overlap between GLGC and FinnGen through FINRISK, Finnish twin cohorts, and NFBC1986.
		a)	Setting: Describe the study design and the underlying population, if possible. Describe the setting, locations, and relevant dates, including periods of recruitment, exposure, follow-up, and data collection, when available.	4–6	Public GWAS summary data were used; no new participants were recruited (§2.1). GLGC 2013 exposures are European-ancestry. FinnGen R11 and UK Biobank BIG40 are identified.
		b)	Participants: Give the eligibility criteria, and the sources and methods of selection of participants. Report the sample size, and whether any power or sample size calculations were carried out prior to the main analysis	5–6; Tables 1–2	The largest marker-specific exposure N and FinnGen case/control counts are reported. Seven endpoints were selected from 2,440 FinnGen phenotypes
		c)	Describe measurement, quality control and selection of genetic variants	6–7	§2.2 specifies $P < 5 \times 10^{-8}$, $F > 10$, GRCh37 positions, European 1000 Genomes Phase 3 reference (n = 503), ≥95% nonmissing genotypes, and 10-Mb LD clumping at $r^2 < 0.001$. Allele harmonization and exclusion of ambiguous palindromic SNPs are described.
		d)	For each exposure, outcome, and other relevant variables, describe methods of assessment and diagnostic criteria for diseases	5–6; Tables 1–2	GLGC 2013 lipid residuals were covariate-adjusted and inverse-normal transformed. FinnGen endpoint codes, diagnostic codes, case/control counts, and the composite stroke and registry memory-symptom definitions are reported (§2.1).

Item No.	Section		Checklist item	Page No.	Relevant text from manuscript
		e)	Provide details of ethics committee approval and participant informed consent, if relevant	16	Institutional Review Board Statement and Informed Consent Statement: “Not applicable.” Only publicly available summary statistics were analyzed (§2.1).
5	**Assumptions**		Explicitly state the three core IV assumptions for the main analysis (relevance, independence and exclusion restriction) as well assumptions for any additional or sensitivity analysis	6–9, 15	The three IV assumptions are listed in §4.4, rather than explicitly set out as the main-analysis assumptions in Methods. The weighted-median ≥50% valid-weight condition and single-causal-signal assumption for ABF colocalization are described in Methods.
6	**Statistical methods: main analysis**		Describe statistical methods and statistics used	6–9	§§2.2–2.5 describe harmonization, multiplicative random-effects IVW, Wald ratios for one instrument, FDR across 28 tests, regional estimates and Q comparisons, weighted median, MR-Egger intercept, and leave-one-out analyses.
		a)	Describe how quantitative variables were handled in the analyses (i.e., scale, units, model)	5, 7	Exposure effects are on a standardized inverse-normal scale; the manuscript describes an MR estimate per 1-SD increase rather than per mg/dL or mmol/L (§2.1). Outcome effects are presented as ORs with 95% CIs (§2.5).
		b)	Describe how genetic variants were handled in the analyses and, if applicable, how their weights were selected	6–8	Instruments were LD-clumped, harmonized, and inverse-variance weighted (§2.2). Instruments were split by a prespecified chr19:44.0–46.5 Mb (GRCh37) APOE interval (§2.4).
		c)	Describe the MR estimator (e.g. two-stage least squares, Wald ratio) and related statistics. Detail the included covariates and, in case of two-sample MR, whether the same covariate set was used for adjustment in the two samples	5–8	GLGC contributing cohorts adjusted for age, age squared, and sex; §2.2 describes multiplicative random-effects IVW and single-SNP Wald ratios.
		d)	Explain how missing data were addressed	6–7, 9	For reference-panel LD estimates, variants with <95% nonmissing genotypes were removed and remaining missing genotypes mean-imputed (§2.2). Unavailable/ambiguous alleles were excluded in harmonization; §2.6 describes exclusions of incompatible and palindromic IDP variants.
		e)	If applicable, indicate how multiple testing was addressed	7, 9	Benjamini–Hochberg FDR adjustment was applied to 28 primary MR tests (§2.2). Thirteen IDP-association tests used Bonferroni adjustment (§2.6). No formal FDR correction was applied to the 26 imaging colocalization comparisons.
7	**Assessment of assumptions**		Describe any methods or prior knowledge used to assess the assumptions or justify their validity	6–9, 12–13	F > 10 was specified for instrument relevance. Cochran Q, MR-Egger intercept, weighted median, and leave-one-out were used to probe heterogeneity/pleiotropy and influence (§§2.2, 2.5; §3.4)

Item No.	Section		Checklist item	Page No.	Relevant text from manuscript
8	**Sensitivity analyses and additional analyses**		Describe any sensitivity analyses or additional analyses performed (e.g. comparison of effect estimates from different approaches, independent replication, bias analytic techniques, validation of instruments, simulations)	7–9	§§2.3–2.6 describe 95 colocalization windows, APOE-region exclusion, weighted median, MR-Egger intercept, leave-one-out, and exploratory BIG40 pairwise colocalization. MR-PRESSO, radial MR, multivariable MR, and multi-signal colocalization were not performed.
9	**Software and pre-registration**				
		a)	Name statistical software and package(s), including version and settings used	9	§2.7 reports Python 3.12.4 and versions of NumPy, pandas, SciPy, statsmodels, Matplotlib, seaborn, requests, openpyxl, and Patsy. Other statistical software mentioned in the Acknowledgments is not specified in §2.7.
		b)	State whether the study protocol and details were pre-registered (as well as when and where)	Not performed	Not performed
	**RESULTS**				
10	**Descriptive data**				
		a)	Report the numbers of individuals at each stage of included studies and reasons for exclusion. Consider use of a flow diagram	5–7; Tables 1–2	Tables 1–2 give exposure N and outcome case/control N. §2.2 reports 303 LD-clumped trait-specific SNP records and 292 harmonized records per outcome (82/75/82/53 by trait).
		b)	Report summary statistics for phenotypic exposure(s), outcome(s), and other relevant variables (e.g. means, SDs, proportions)	5–6; Tables 1–2	Largest marker-specific exposure sample sizes and outcome case/control counts are reported.
		c)	If the data sources include meta-analyses of previous studies, provide the assessments of heterogeneity across these studies	5	The exposures come from a GLGC 2013 meta-analysis (§2.1)
		d)	For two-sample MR:<br>i. Provide justification of the similarity of the genetic variant-exposure associations between the exposure and outcome samples<br>ii. Provide information on the number of individuals who overlap between the exposure and outcome studies	5–6, 15	The manuscript states that GLGC 2013 and FinnGen R11 may overlap at the cohort level through FINRISK, Finnish twin cohorts, and NFBC1986 (§2.2). 'GWAS_inventory_and_sample_overlap.xlsx' inventory workbook documents this potential overlap.
11	**Main results**				
		a)	Report the associations between genetic variant and exposure, and between genetic variant and outcome, preferably on an interpretable scale	6–7; Table A1	§2.2 reports per-SNP exposure instruments and corresponding Wald ratios; the 303 LD-clumped variants are supplied in Supplementary_Table_SNP_instruments.xlsx.

Item No.	Section		Checklist item	Page No.	Relevant text from manuscript
		b)	Report MR estimates of the relationship between exposure and outcome, and the measures of uncertainty from the MR analysis, on an interpretable scale, such as odds ratio or relative risk per SD difference	10; Table A1	§3.1 and Table A1 report IVW ORs, 95% CIs, P values, and FDR-adjusted P values for all 28 lipid–outcome combinations.
		c)	If relevant, consider translating estimates of relative risk into absolute risk for a meaningful time period	Not applicable	The study reports genetic MR odds ratios from summary data, without a baseline absolute risk over a defined follow-up period.
		d)	Consider plots to visualize results (e.g. forest plot, scatterplot of associations between genetic variants and outcome versus between genetic variants and exposure)	10–12; Figures 2, 4	Figure 2 presents MR estimates across outcomes, and Figure 4 presents APOE-region versus non-APOE-region estimates.
12	**Assessment of assumptions**				
		a)	Report the assessment of the validity of the assumptions	12–13, 15	§3.4 reports MR-Egger intercept, weighted-median and leave-one-out summaries; §4.4 discusses pleiotropy, lipid-trait overlap, and the limitations of MR assumptions.
		b)	Report any additional statistics (e.g., assessments of heterogeneity across genetic variants, such as I^2, Q statistic or E-value)	12–13, 21–22	Cochran Q heterogeneity is summarized in §3.4 (19/28 full-instrument and 14/28 non-APOE analyses); regional Q comparisons are reported in Table A3.
13	**Sensitivity analyses and additional analyses**				
		a)	Report any sensitivity analyses to assess the robustness of the main results to violations of the assumptions	12–13, 21–24	§3.4 compares IVW and weighted-median estimates and reports MR-Egger intercept and leave-one-out summaries. Figures A1–A2 present sensitivity and leave-one-out plots; complete numerical leave-one-out results are supplied in Supplementary_Table_LOO_results.xlsx.
		b)	Report results from other sensitivity analyses or additional analyses	11–13; Tables A2–A4	APOE-region exclusion and colocalization results are reported (§§3.2–3.5). Table A2 contains 95 regional analyses; Table A4 contains the 26 HDL-C/stroke–IDP pairwise analyses.
		c)	Report any assessment of direction of causal relationship (e.g., bidirectional MR)	Not performed	No reverse-direction or bidirectional MR analysis is described. Direction of the causal relationship was not assessed separately.
		d)	When relevant, report and compare with estimates from non-MR analyses	2, 14–15	The Introduction and Discussion compare findings with prior observational and genetic literature.
		e)	Consider additional plots to visualize results (e.g., leave-one-out analyses)	23–24	Figures A1 and A2 present sensitivity and leave-one-out plots; Figure 5 presents HDL-C–stroke sensitivity estimates.
	**DISCUSSION**				

Item No.	Section		Checklist item	Page No.	Relevant text from manuscript
14	**Key results**		Summarize key results with reference to study objectives	13–16	§4 summarizes the outcome-specific findings, APOE-region sensitivity, and exploratory non-APOE imaging results; §5 restates the principal findings.
15	**Limitations**		Discuss limitations of the study, taking into account the validity of the IV assumptions, other sources of potential bias, and imprecision. Discuss both direction and magnitude of any potential bias and any efforts to address them	15–16	§4.4 discusses pleiotropy, shared determinants of lipid traits, lifelong genetic exposure versus clinical treatment, broad registry outcomes, few regional instruments, and the single-signal colocalization assumption.
16	**Interpretation**				
		a)	Meaning: Give a cautious overall interpretation of results in the context of their limitations and in comparison with other studies	14–16	§§4.1–4.4 compare prior studies, qualify APOE-region analyses as sensitivity results, and avoid treating pairwise imaging colocalization as mediation. Conclusions distinguish HDL-C–stroke from the APOE-sensitive AD/dementia estimates.
		b)	Mechanism: Discuss underlying biological mechanisms that could drive a potential causal relationship between the investigated exposure and the outcome, and whether the gene-environment equivalence assumption is reasonable. Use causal language carefully, clarifying that IV estimates may provide causal effects only under certain assumptions	15–16	§4.3 discusses ILRUN and explicitly says the imaging overlap does not identify a causal variant, effector gene, or mediating pathway. §4.4 notes that lifelong genetically proxied differences should not be equated with treatment effects.
		c)	Clinical relevance: Discuss whether the results have clinical or public policy relevance, and to what extent they inform effect sizes of possible interventions	15–16	§4.4 states that MR estimates of lifelong genetic differences should not be equated with the effects of treatment initiated at a particular age.
17	**Generalizability**		Discuss the generalizability of the study results (a) to other populations, (b) across other exposure periods/timings, and (c) across other levels of exposure	5, 15–16	The exposure GWAS comprised European-ancestry participants. §4.4 notes that lifelong genetically proxied differences cannot be directly translated into later-life interventions.
	**OTHER INFORMATION**				
18	**Funding**		Describe sources of funding and the role of funders in the present study and, if applicable, sources of funding for the databases and original study or studies on which the present study is based	16	The Funding statement lists three institutional funding sources and grant numbers.
19	**Data and data sharing**		Provide the data used to perform all analyses or report where and how the data can be accessed, and reference these sources in the article. Provide the statistical code needed to reproduce the results in the article, or report whether the code is publicly accessible and if so, where	16	The Data Availability Statement links to GLGC 2013, FinnGen R11, Oxford BIG40, and GWAS Catalog and provides the project-code URL. The accompanying supplements include GWAS_inventory_and_sample_overlap.xlsx, Supplementary_Table_SNP_instruments.xlsx, Supplementary_Table_LOO_results.xlsx, and the other analysis tables. §2.7 states that complete analysis scripts are supplied with the reproducibility materials.

Item No.	Section	Checklist item	Page No.	Relevant text from manuscript
20	**Conflicts of Interest**	All authors should declare all potential conflicts of interest	16	The Conflicts of Interest statement reads: “The authors declare no conflicts of interest.”

This checklist is copyrighted by the Equator Network under the Creative Commons Attribution 3.0 Unported (CC BY 3.0) license.

1. Skrivankova VW, Richmond RC, Woolf BAR, Yarmolinsky J, Davies NM, Swanson SA, et al. Strengthening the Reporting of Observational Studies in Epidemiology using Mendelian Randomization (STROBE-MR) Statement. JAMA. 2021;under review.

2. Skrivankova VW, Richmond RC, Woolf BAR, Davies NM, Swanson SA, VanderWeele TJ, et al. Strengthening the Reporting of Observational Studies in Epidemiology using Mendelian Randomisation (STROBE-MR): Explanation and Elaboration. BMJ. 2021;375:n2233.
